# MIRABEL: a research program to develop tools for risk and cost/benefit analysis of food allergens

**DOI:** 10.1186/2045-7022-1-S1-O6

**Published:** 2011-08-12

**Authors:** Amélie Crépet, Fanny Héraud, Stéphan Marette, Moneret-Vautrin Denise-Anne, Jutta Roosen

**Affiliations:** 1 ANSES, Maisons-Alfort, France; 2 INRA, Thiverval–Grignon, France; 3 Allergo-vigilance Network, Vandoeuvre Les Nancy, France; 4 TUM, Munich, Germany

## 

Allergenic foods represent a significant health risk for persons who have an allergy to specific food. However, this risk remains poorly characterised. Knowledge is often missing on the different risk components such as the presence of allergens in food, food consumption behaviours of allergic sufferers and thresholds of reaction. Usual risk assessment approaches cannot be easily implemented to assess the risk from sporadic contamination and that concerns only a small part of the population. As a consequence, there is a lack of risk-based guidance and operational tools of those involved in allergen risk management, who are food industries, the allergic individuals and the food regulators. To upgrade knowledge, methodological and operational tools in food allergy, MIRABEL involves various scientific fields such as chemical analysis, dietary survey, medical research, socio-economics, applied mathematics and statistics to exposure and risk assessments. In this way, MIRABEL aims to set an integrated and operational framework for the allergic risk analysis, in order to improve quality of life of allergic sufferers. Each risk component will be investigated to accurately characterize the risk of food allergy and to test different risk management options. To complete existing but sparse information, field surveys will be conducted in order to acquire accurate data on allergic consumers’ behaviours relating to allergen-containing products and their thresholds of reaction, and to allergen presence in food consumed by allergic sufferers. In parallel, methodological developments in Bayesian statistics and probabilistic modelling will be realized to be able to combine the acquired data in an integrated risk quantification model. A cost-benefits analysis for the stakeholders (food industry, allergic individuals and regulators) will also be conducted to anticipate impacts of new policies. The project will be focused on the peanut, which can be adventitiously present in various foodstuffs such as chocolate, cereals or biscuits. This allergen is associated with the highest prevalence of food allergy and is one of the food allergens that lead to the most severe adverse effects.

